# An audit cycle of physical health monitoring and record keeping of long term in-patients at male and female psychiatric rehabilitation wards using QI approach

**DOI:** 10.1192/bjo.2021.318

**Published:** 2021-06-18

**Authors:** Hina Tahseen, Peter Bramall

**Affiliations:** Delfryn Lodge, Cygnet Health Care

## Abstract

**Aims:**

To complete an audit cycle to evaluate and improve physical health monitoring practice for in-patients by incorporating small QI based projects between baseline audit and re-audit.

**Background:**

People with mental health illness are at increased risk of physical illness, morbidity and mortality compared with general population, mainly due to adverse effects of psychotropic medications, polypharmacy, poor lifestyle choices and socio-economic difficulties. It is important to recognise the need for active health promotion, including formal health checks for psychiatric in-patients.

**Method:**

Standards were obtained from NICE Guidelines, RCPsych Report on Physical Health in Mental Health and Cygnet Health Care's Physical health policy.

An Audit tool with simple checklist was generated from key areas of Cygnet's physical health policy. Physical Health Files of 24 patients from Female Rehabilitation Ward and 28 patients from Male Rehabilitation Ward were audited in the initial audit cycle.

Checklist included physical health examination within 24 hours of admission, Annual Health Improvement Profile (HIP), Monthly physical health reviews (including observations and weights), High Dose Antipsychotics Monitoring, Bloods and ECG records. After the initial baseline audit in Apr., 2019, some of the Quality Improvement (QI) approaches (4 PDSA cycles, driver diagrams, model for improvement) were used before conducting the re-audit in Oct., 2019.

**Result:**

The baseline audit in Apr., 2019 showed 98% compliance with physical assessment within 24 hours of admission, however, there was a significant gap in the monthly physical health reviews (62%), Annual HIP (30%), High-dose antipsychotic monitoring (10%) and ECG/Bloods for antipsychotic monitoring (64%) as per guidelines. 10 Female and 12 male patients had regularly refused obs, weight checks and physical health monitoring.

The re-audit showed an overall improvement of 92% in compliance, with increased High-dose antipsychotic monitoring (100%), Monthly physical health clinics (88%), Annual HIP (75%), Annual antipsychotic monitoring/bloods/ECG(95%).

**Conclusion:**

Interventions, using QI approaches, between baseline and re-audit, included MDT discussion around strategies to improve patients’ engagement with monthly physical health clinics with Specialty doctor, adding to care plan points, timescales and reminders in doctors’ diaries for next bloods and ECGs due, MDT and patients’ health education and a designated support staff for physical obs and maintaining physical health files. This helped in providing a framework to test recommended changes and evolve design based on repeated date collection between cycles.

The QI Interventions helped in implementation of a more holistic approach towards assessments due to which, the re-audit demonstrated a sustained improvement in compliance with all aspects of physical health monitoring.

